# Suicide ideation and the related factors among Iranian transgender people: a cross-sectional study

**DOI:** 10.1186/s13104-023-06471-3

**Published:** 2023-09-04

**Authors:** Taranom Arianmehr, Younes Mohammadi

**Affiliations:** 1 Department of Epidemiology, School of Public Health, Hamadan University of Medical Sciences, Hamadan, Iran; 2 Students Research Committee, Hamadan University of Medical Sciences, Fahmideh Street, Hamadan, Iran

**Keywords:** Suicide ideation, Gender identity disorder, Transgender, Iran

## Abstract

**Objective:**

Suicide is a significant public health concern worldwide, and efforts to prevent it are crucial. This study aims to assess suicide ideation among transgender individuals in Iran. A cross-sectional design was used, with 235 transgender participants recruited through snowball sampling across Iran. The data collection included a questionnaire containing demographic inquiries, while the Beck Scale for Suicide Ideation was employed to gather data. Descriptive and analytical statistics were performed using SPSS software.

**Results:**

The findings indicate that the prevalence of suicidal ideation among the transgender individuals in the study was 83%, with a mean score of 12.8 ± 8.8 on the suicide ideation scale. Individuals with lower levels of education, younger age, unemployment, and being divorced or single demonstrated significantly higher scores compared to others (p < 0.05). This study highlights the high prevalence of suicidal ideation among Iranian transgender individuals, particularly among those with lower age and education levels, as well as those who have not undergone gender reassignment surgery.

## Introduction

Suicide is a critical global health issue, with the World Health Organization reporting over 800,000 suicide deaths annually [1]. In addition to mortality cases, the incidence of non-fatal suicide attempts was found to be 20 times higher than the number of completed suicides resulting in mortality [2]. A range of factors, including biological, psychological, familial, socioeconomic, and cultural circumstances, contribute to the risk of suicide [3–6]. Suicide ideation, characterized by persistent thoughts of suicide or self-harm, is the starting point for death by suicide and is considered an emergency issue in psychiatry [7]. Early identification, prevention, and treatment of suicide ideation are essential to reducing the risk of suicide and promoting mental wellness [8, 9].

Transgender individuals are those whose gender identity differs from the gender assigned to them at birth [10]. Across all societies and throughout their lives, they face numerous social and psychiatric challenges. Studies have consistently shown that trans individuals experience higher levels of discrimination compared to their cisgender counterparts, both in society and within their own families [11, 12]. Moreover, research has indicated that transgender individuals worldwide have a higher prevalence of suicide ideation and suicide attempts [13–16]. However, in the Iranian community, cultural and religious conditions have resulted in a lack of research and understanding regarding issues related to transgender people [17]. As a result, our knowledge about the health status of transgender individuals, including suicide ideation, is limited. Therefore, it is critical to design studies that can provide insights into the health status of this population.

Aim of this study is to explore suicide ideation among Iranian transgender individuals and investigate some of the factors associated with it. By conducting this research, we hope to contribute to the understanding of the mental health challenges faced by this marginalized population.

## Method

### Study design and setting

This cross-sectional study was conducted in 2020 across five major cities in Iran - Tehran, Mashhad, Esfahan, Shiraz, and Karaj - in collaboration with the Iranian Association for the Support of Transgender People (abbreviated MELAAL in Persian).

### Study population

The study population consisted of transgender individuals. We accessed the participant pool through a combination of face-to-face interactions, specifically through MELAAL, as well as through respective transgender groups on social media platforms, such as Instagram and Telegram. Inclusion criteria included individuals who identify as trans women (assigned male at birth but identify as female) or trans men (assigned female at birth but identify as male). Exclusion criteria consisted of lesbian, gay and heterosexual individuals.

### Sampling technique

Given the cultural limitations and negative perspectives surrounding transgender people in Iran, access to Iranian transgender individuals is challenging. Therefore, we employed a snowball sampling technique to recruit participants for this study. Initially, we contacted key informative transgender individuals in MELAAL and requested their assistance in introducing us to social groups comprised of transgender individuals. Within these groups, after completion of the questionnaire, we again requested introductions to additional transgender individuals or groups throughout Iran.

### Sample size

Due to the nature of our study design, which utilized snowball sampling for participant recruitment within the transgender community, it was not feasible to pre-determine a specific sample size. Snowball sampling is commonly employed in studies involving hard-to-reach or marginalized populations, such as transgender individuals, where participant recruitment can be challenging and uncertain. However, the final sample size obtained for this study was 235 transgender participants.

### Data collection tools

To collect data for this study, we employed a valid and reliable questionnaire comprising two segments. The first segment included demographic questions, such as age, gender at birth, education level, and history of gender reassignment surgery. As for the second segment, we utilized the Beck Questionnaire, developed by Beck [18], to measure suicide ideation. The validity and reliability of Iranian version of the questionnaire have been confirmed before [19]. The eigenvalue of this factor was 2.98, and it accounted for 59.5% of the total variance. Moreover, Cronbach’s alpha coefficients for this questionnaire was reported 80%.

The Beck Questionnaire was completed via self-administration by study participants. They were provided with the questionnaire and asked to complete it to the best of their ability.

### Ethics statement

This study has received approval from the Ethics Committee of Hamadan University of Medical Sciences (IR.UMSHA.REC.1398.781). Furthermore, informed written consent was obtained from all participants before completing the questionnaire.

### Statistical analysis

Descriptive statistics, including mean and standard deviation for quantitative data, and frequency and percentage for qualitative data, were employed. Additionally, independent t-tests, analysis of variance (ANOVA), and chi-square tests were utilized to determine the significance of associations.

## Results

Table 1 presents the demographic characteristics of 235 transgender individuals from various regions in Iran. The average age of the participants was 24.2 ± 4.8 years, with the majority identifying as trans men (80.4%). Furthermore, 96% of the participants were single, and only 5% had undergone gender reassignment surgery.

**Table 1:** Demographic characteristics of the trans individuals participated in this study

variables		frequency	Percentage
Gender	Trans Man	189	80.4
	Trans Woman	46	19.6
Gender Reassignment Surgery	Yes	13	5.5
	No	222	94.5
Education	Middle School	14	5.96
	High School	75	31.91
	Diploma	84	35.74
	Associate Degree	23	9.79
	Bachelor	33	14.04
	Master	6	2.55
Work Status	Employed	48	20.43
	University Student	66	28.09
	Student	82	34.89
	Jobless and Housewife	39	16.60
Marital Status	Single	226	96.17
	Married	4	1.70
	Divorced	5	2.13
Suicidal Ideation	Yes No	195 40	83 17

As shown in Table 2, the average score for suicide ideation among transgender individuals was 12.8 ± 8.8. Table 2 also displays the average scores for suicide ideation based on each demographic variable. It is evident that there are differences at the variable levels. With the exception of gender, all variables showed a significant association with suicide ideation. Transgender individuals who underwent gender reassignment surgery had significantly lower scores of suicide ideation compared to those who did not undergo the procedure (P = 0.03). Regarding marital status, singles had the highest score of suicidal ideation (13 ± 8.8), while married individuals had the lowest score (4.5 ± 01.7), which was statistically significant (p = 0.04).

**Table 2:** Mean and standard deviation of suicide ideation scores by demographic variables

variables	p-value		
Gender	Trans Man	12.4 ± 8.6	0.94
	Trans Woman	12.9 ± 8.1	
Gender Reassignment Surgery	Yes	3.9 ± 4.1	0.029
	No	13.3 ± 8.7	
Marital Status	Single	13 ± 8.8	0.04
	Married	4.5 ± 1.73	
	Divorced	10 ± 7.5	
Education Level	Middle School	17.5 ± 9.4	0.001
	High School	15.3 ± 9.1	
	Diploma	12.1 ± 8.6	
	Associate’s Degree	8.9 ± 7.7	
	Bachelor	11 ± 7.4	
	Master	5.7 ± 3.3	
Age Groups			0.002
	Under 20 years	16.3 ± 8.9	
	21 to 25 years	10.4 ± 7.1	
	26 to 30 years	6.7 ± 4.3	
	31 to 35 years	4.2 ± 5.5	
	36 to 44 years	5 ± 01.7	
Work Status	Employed	8.1 ± 7.5	< 0.001
	University Student	12.3 ± 9.1	
	Student	16 ± 8.9	
	Unemployed	12.6 ± 6.8	

Furthermore, an analysis by age groups revealed an inverse relationship between age and the score of suicidal ideation. The younger age groups had the highest scores, while the older age groups had the lowest scores, and this difference was statistically significant (p = 0.002). The different employment statuses were associated with varying scores of suicidal ideation. Employed individuals had the lowest score (8.1 ± 7.5), while students had the highest score (16.02 ± 8.9), as confirmed by ANOVA testing, which showed a significant association (p < 0.0001).

Statistical analysis also indicated a significant difference in suicidal ideation scores among individuals with different levels of education. Those with lower education levels had higher scores, whereas individuals with higher levels of education had lower scores (p < 0.001). However, the mean scores of suicidal ideation for trans men and trans women were comparable and statistically not significant (p = 0.94).

Table 3 presents the results of the regression analysis, examining the association between variables and suicidal ideation. In this model, education variable was considered as dummy variable. The table reveals that after adjusting for other variables, only two factors, age and gender assignment surgery, were statistically significant, while the other variables did not have a significant effect. Furthermore, the standardized Beta results indicated that age had the most significant influence on suicidal ideation, followed by gender assignment surgery.

**Table 3:** Results of linear regression for association of the variables with suicidal ideation

Variables	Beta	Std. Error	Standardized Beta	
(Constant)	25.7	3.3		0.000
age	− 0.54	0.16	− 0.29	0.001
Gender at birth	1.4	1.4	0.06	0.32
surgery	-6.4	2.4	-0.17	0.009
marital	0.092	1.8	0.003	0.96
High School	-1.17	2.4	-0.06	0.63
Diploma	-2.3	2.5	-0.13	0.35
Associate’s Degree	-3.99	2.99	-0.13	0.18
Bachelor	-2.2	2.83	-0.09	0.42
Master	-2.5	4.5	-0.04	0.59

## Discussion

The present study investigated suicide ideation among transgender individuals in Iran. The results revealed a relatively high score of suicide ideation among Iranian transgender individuals (12.8 ± 8.8). Notably, gender reassignment surgery was found to be associated with suicide ideation. Transgender individuals who underwent gender reassignment surgery had significantly lower scores of suicide ideation compared to those who did not undergo the procedure. This finding is supported by previous studies that reported lower suicide ideation levels in trans individuals who received medical interventions such as hormone therapy or gender reassignment surgery [16, 18].

Furthermore, age was not significantly associated with suicide ideation among the transgender individuals in our study (p = 0.324). However, other studies have reported a significant relationship between age and suicide ideation, indicating that as age increases, readiness for suicide decreases [15, 18].

Additionally, though there were varying scores of suicide ideation based on educational levels in our study, this difference was not statistically significant. In contrast, some researchers have reported that high school education or its equivalent is linked to an increased risk of suicide in transgender individuals [20]. The discrepancy in findings may be attributed to the relatively small sample size in our study.

Previous research exploring the relationship between gender reassignment surgery and the rate of suicide ideation in transgender individuals has typically focused on specific populations, such as clients of organizations or specialist physicians in particular provinces. For instance, Mahmoodi (2019) assessed suicide ideation in 60 transgender individuals chosen from the clients of the Iran Association of Gender Identity Disorder [21]. The study reported a significantly lower mean score of suicide ideation in individuals who underwent sex reassignment surgery compared to those who did not (10.56 vs. 16.26). Similarly, Asadipour et al. (2019) investigated the frequency of suicide ideation in 70 trans men in Fars Province and found that those who underwent gender reassignment surgery had a lower score of suicide ideation compared to those without the surgery (P < 0.05) [20].

Aghabakhshi (2009) conducted interviews with 21 transgender individuals and identified various factors contributing to suicide ideation. The study highlighted family factors such as neglect, ridicule, forced traditional gender clothing, and physical abuse as some of the major causes of suicide ideation in trans individuals [22]. These factors, along with others such as sexual dissatisfaction, failure in love and marriage, family and social rejection, discrimination, and shame, can lead to suicide ideation due to the unconventional gender identity of trans individuals [23–25]. Gender reassignment surgery may alleviate some of these problems, increasing trans people’s satisfaction with life and reducing suicide ideation. It can potentially enhance sexual satisfaction, enable the possibility of marriage, and mitigate social stigma and rejection.

This study has several limitations that should be considered when interpreting the results. First, due to cultural stigma and negative perspectives towards transgender individuals in Iran, snowball sampling was utilized for recruitment. While necessary given the marginalized study population, this sampling method may introduce bias and limit the generalizability of the findings beyond the sampled social networks. Second, data on other factors empirically associated with suicide ideation, such as mental health issues, substance use, and experiences of discrimination, were not collected. Controlling for these variables could have provided a more comprehensive understanding of suicide ideation risks. Third, an a priori sample size calculation was not performed, which may have resulted in inadequate statistical power to detect smaller effects or subgroup differences. The convenience sample of 235, though feasible, may have been underpowered based on the effect sizes found. Future studies should perform power analyses using effect size estimates from the literature or pilot data to determine target sample sizes. Finally, this study was conducted during the COVID-19 pandemic, which could have influenced mental health outcomes, limiting generalizability to non-pandemic conditions.

Despite these limitations, this study provides valuable preliminary insights on factors associated with suicide ideation in a vulnerable transgender population that can inform future research.

## Conclusion

The study reveals alarming results regarding the mental health of transgender individuals in Iran. Suicidal ideation is highly prevalent among this population, especially among young people, those with low levels of education, and those who have not undergone gender reassignment surgery. These findings highlight the urgent need to address the mental health concerns of transgender individuals in Iran and provide them with adequate support and resources. Failure to do so could lead to severe consequences and further exacerbate the already challenging situation faced by this vulnerable group.

## Data Availability

The data that support the findings of this study are available from the corresponding author, upon reasonable request.
